# Supplementary material for: In situ copper faceting enables efficient CO2/CO electrolysis
Source: Nat Commun. 2024 Feb 26;15:1749. doi: 10.1038/s41467-024-45538-y (PMC10897386; doi:10.1038/s41467-024-45538-y)
Supplement: Supplementary file 1 — Supplementary Information [file 41467_2024_45538_MOESM1_ESM.pdf]

Supporting Information for

In situ copper faceting enables efficient CO_2/CO electrolysis

Kaili Yao,[1, 2, †], *Jun Li,*[3, †,] *Adnan Ozden,[4, †] *Haibin Wang,*[1, †] *Ning Sun,*[3] *Pengyu Liu,*[3] *Wen Zhong,*[3] *Wei Zhou,*[5] *Jieshu Zhou,*[1] *Xi Wang,*[1] *Hanqi Liu,*[3] *Yongchang Liu,*[1,6] *Songhua Chen,*[7] *Yongfeng Hu,*[8] *Ziyun Wang,*[9] *David Sinton,*[4,*] *Hongyan Liang*[1,*]

[†]These authors contributed equally to this work.

[*]Correspondence and requests for materials should be addressed to Jun Li, David Sinton and Hongyan Liang (lijun001@sjtu.edu.cn; sinton@mie.utoronto.ca; hongyan.liang@tju.edu.cn).

Table of Contents

1. Supplementary Figures

Fig. 1 | Model structure of Cu(100) and Cu(111).
Fig. 2 | E^{avg} of *CO and OH^- on Cu(111) and Cu(100) at various coverage.
Fig. 3 | The adsorption structure of Cu(100) with different *CO coverage.
Fig. 4 | The adsorption structure of Cu(100) with different OH^- coverage.
Fig. 5 | The adsorption structure of Cu(111) with different *CO coverage.
Fig. 6 | The adsorption structure of Cu(111) with different OH^- coverage.
Fig. 7 | The work function of Cu(100) with different OH^- coverage.
Fig. 8 | PDOS of Cu atom on Cu(111) and Cu(100) with different*CO and OH^- coverage.
Fig. 9 | P/Cu atomic ratio in the precatalysts measured by EDX and ICP-OES.
Fig. 10 | SEM images of precatalysts.
Fig. 11 | Characterization of the CuP0 precatalyst.
Fig. 12 | Characterization of the CuP0.4 precatalyst.
Fig. 13 | The Cu K-edge XANES derivative spectra of Cu precatalyst and standards.
Fig. 14 | High-resolution XPS spectra of CuP0.4 and CuP0 precatalysts.
Fig. 15 | XRD patterns for precatalysts and corresponding derived Cu catalysts.
Fig. 16 | FTIR spectra for precatalysts.
Fig. 17 | Possible geometry structures of phosphate-copper complexes.
Fig. 18 | High-resolution XPS spectra of the precatalyst and derived Cu-based catalysts.
Fig. 19 | High-resolution and dark-field TEM images for Cu(100)-rich catalyst.
Fig. 20 | Dark-field microscope images and SEM images of CuP0 precatalyst and derived Cu catalysts.
Fig. 21 | High-resolution and dark-field TEM images for Cu catalyst.
Fig. 22 | CV curves for Cu(100)-rich and Cu catalysts and the corresponding facet ratio.
Fig. 23 | A photograph of the *in-situ* Raman setup.
Fig. 24 | Raman spectra of CuP0 and CuP0.4 precatalysts.
Fig. 25 | Time-dependent *in-situ* ATR-SEIRAS spectra for CuP0.4 and CuP0 precatalysts.
Fig. 26 | Time-dependent Cu K-edge XAFS spectra before normalized of CuP0.4 precatalys.
Fig. 27 | Time-dependent Cu K-edge XANES and EXAFS of control CuP0 precatalyst.
Fig. 28 | Performance comparison for the Cu-based catalysts tested in MEA-CO_2R systems.
Fig. 29 | CV curves for Cu and Cu(100)-rich catalyst and ECSA-normalized j_{C2+}.
Fig. 30 | *In-situ* Raman spectra for Cu(100)-rich catalyst derived from CO_2R and COR.
Fig. 31 | Performance comparison for the Cu-based catalysts tested in MEA-COR systems.

2. Supplementary Tables

Table 1 | Surface energies for Cu(100) and Cu(111) facets at different coverages of CO* and OH^-.

Table 2 | The dipole moment of Cu(100) and Cu(111) with different OH^- coverage.
Table 3 | Work function of Cu(100) with 1/9 ML and 2/9 ML of OH^- coverage.
Table 4 | The energy of 1/9 ML of OH^- coverage on Cu(100) with/without spin polarization.
Table 5 | Calculated area proportion of Cu(100) and Cu(111) facets at different coverages of CO* and OH^- from the Wulff construction analysis.
Table 6 | Atomic percentages of elements in precatalysts measured by EDX and ICP-OES.
Table 7 | Comparison of this work with a previous work that growth of Cu(100) using *CO.
Table 8 | The Cu(100)/(Cu(100)+Cu(111)) ratio of different Cu catalysts.
Table 9 | Linear combination fit analysis of CuP0.4 precatalyst at different reduction times.
Table 10 | EXAFS fitting results of CuP0.4 precatalyst at different reduction times.
Table 11 | Performance comparison for the Cu-based catalysts in MEA-CO_2R systems.
Table 12 | Double-layer capacitance and ECSA for Cu and Cu(100)-rich catalysts.
Table 13 | Performance comparison for the Cu-based catalysts in MEA-COR systems.

3. Supplementary References

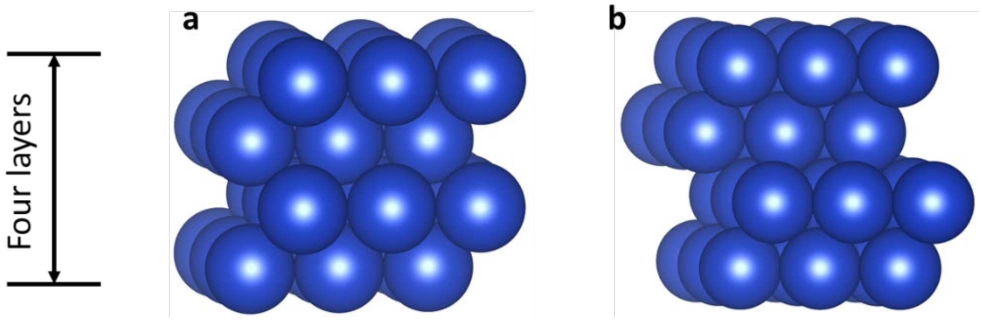

Supplementary Fig. 1 | Model structure of **(a)** Cu(100) and **(b)** Cu(111).

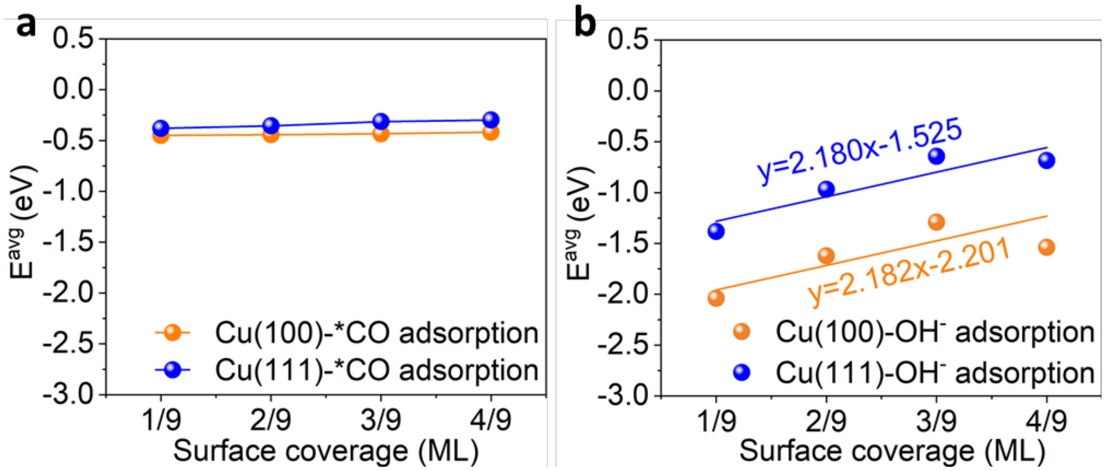

Supplementary Fig. 2 | E^{avg} of **(a)** *CO and **(b)** OH^- on Cu(111) and Cu(100) at various coverage. We exclude the adsorbate-adsorbate interaction as it does not affect the relative trend of surface energies between Cu(100) and Cu(111).

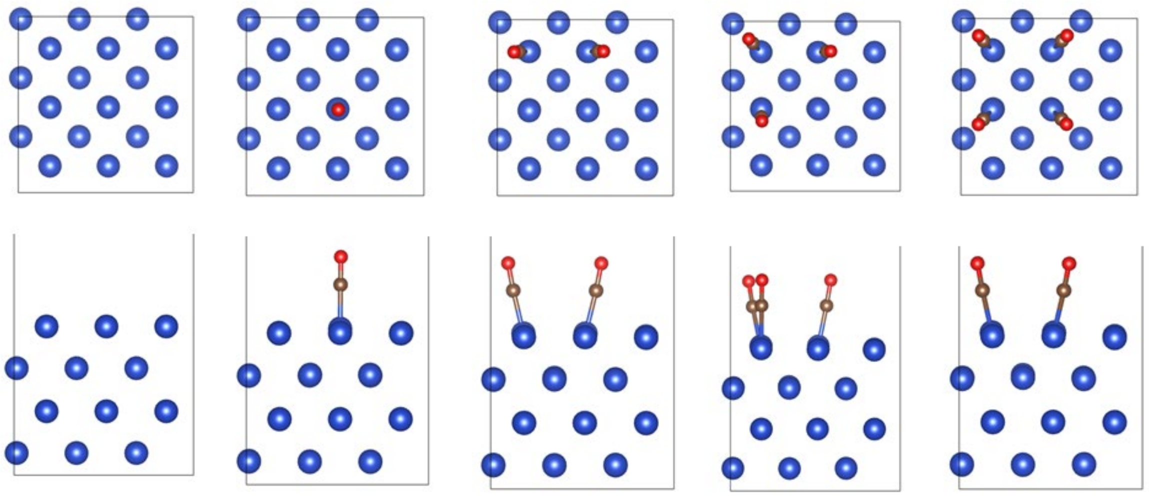

Supplementary Fig. 3 | The top and side views of adsorption structure of Cu(100) with different *CO coverage.

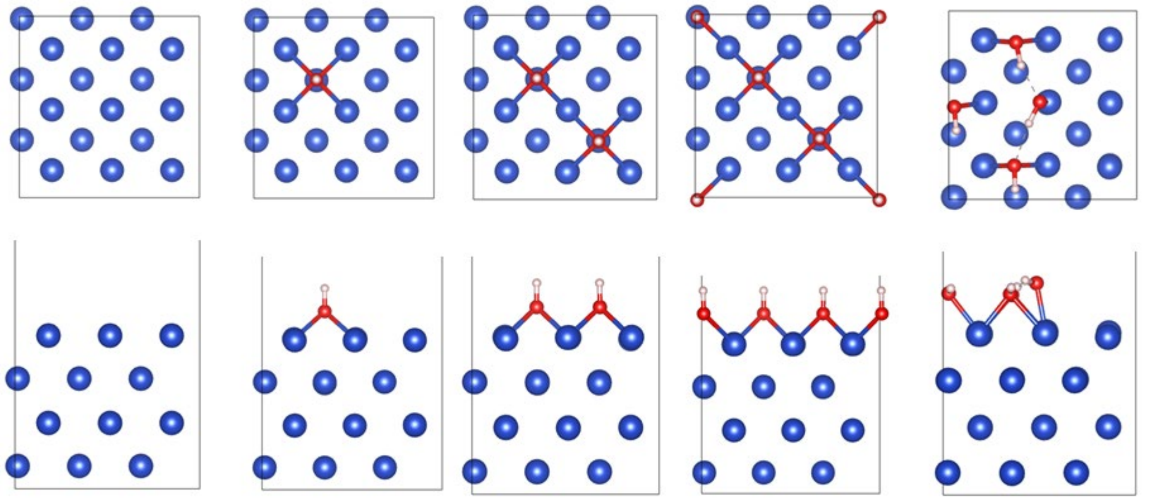

Supplementary Fig. 4 | The top and side views of adsorption structure of Cu(100) with different OH^- coverage.

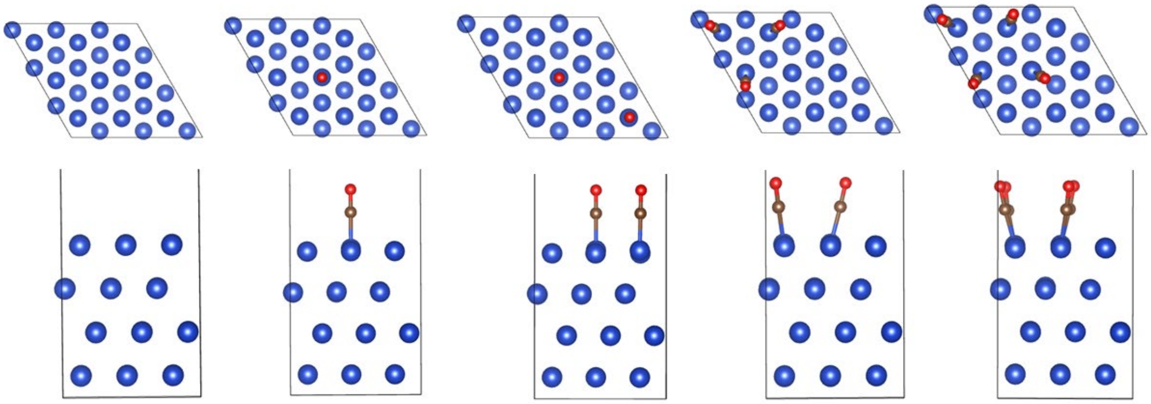

Supplementary Fig. 5 | The top and side views of adsorption structure of Cu(111) with different *CO coverage.

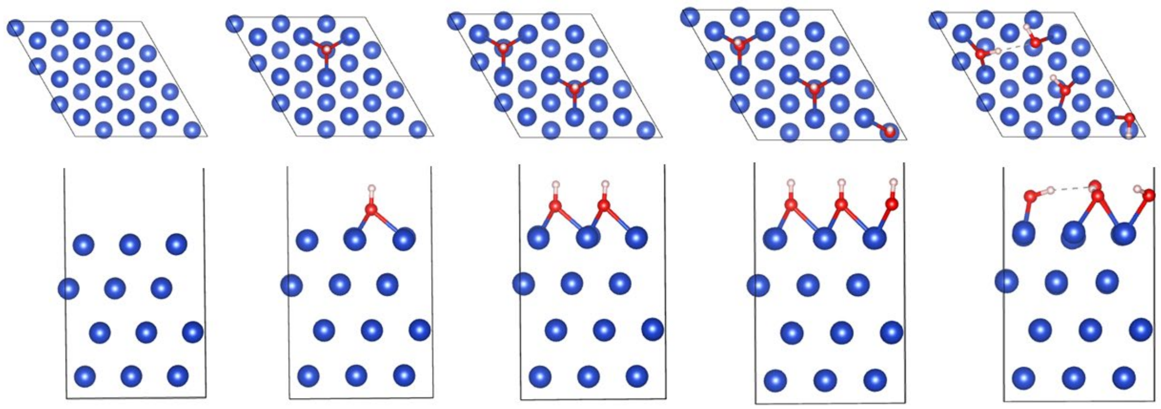

Supplementary Fig. 6 | The top and side views of adsorption structure of Cu(111) with different OH^- coverage.

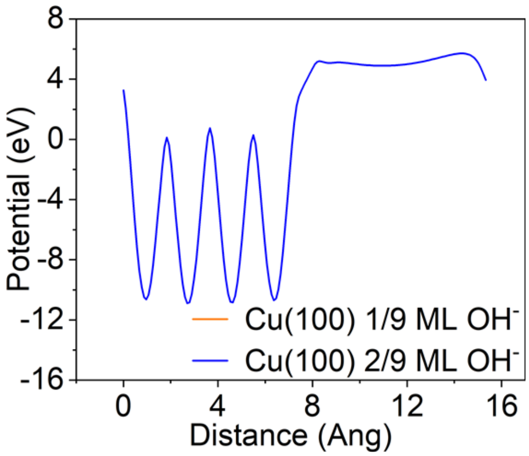

Supplementary Fig. 7 | The work function of Cu(100) with 1/9 ML and 2/9 ML of OH^{-} coverage.

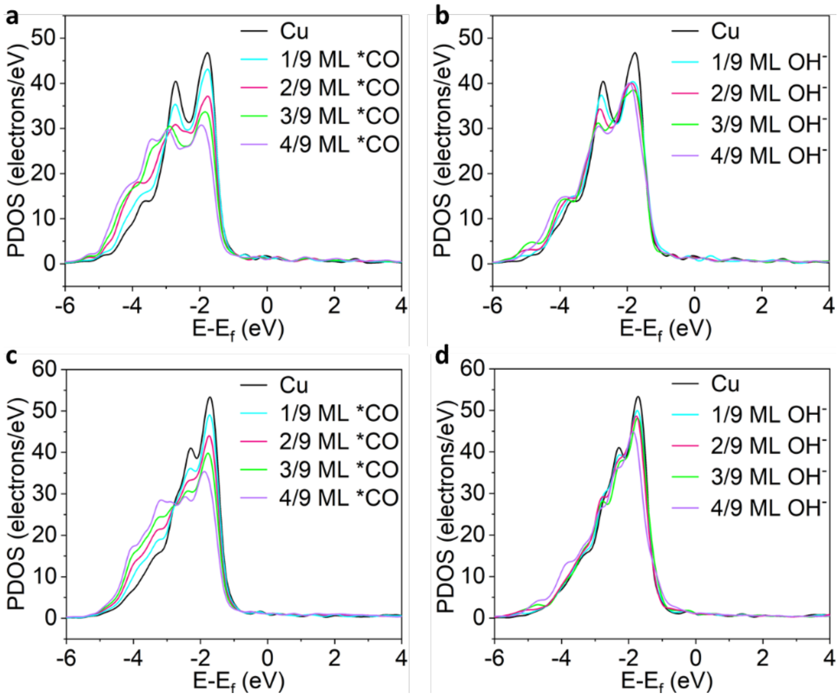

Supplementary Fig. 8 | The projected density of state (PDOS) of top Cu atom on Cu(111) with different **(a)** *CO coverage and **(b)** OH^- coverage; PDOS of top Cu atom on Cu(100) with different **(c)** *CO coverage and **(d)** OH^- coverage.

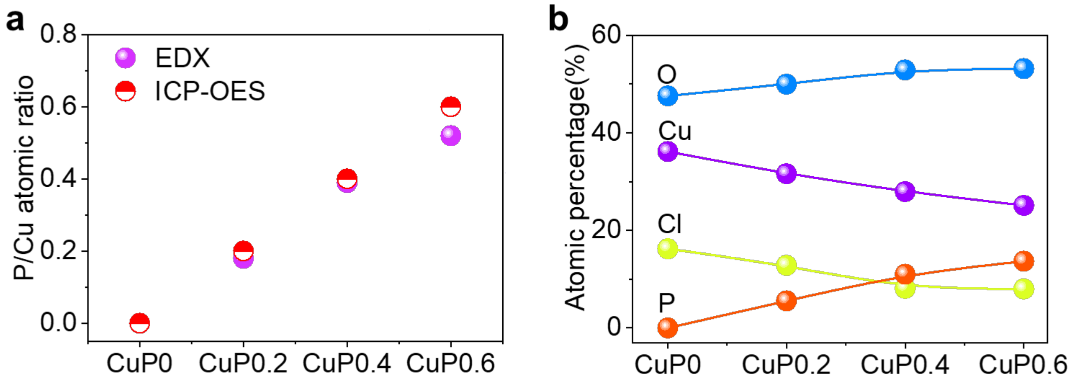

Supplementary Fig. 9 | **a,** P/Cu atomic ratio in the precatalysts measured by EDX and ICP-OES. **b,** The atomic percentages of different elements in the precatalysts measured by EDX.

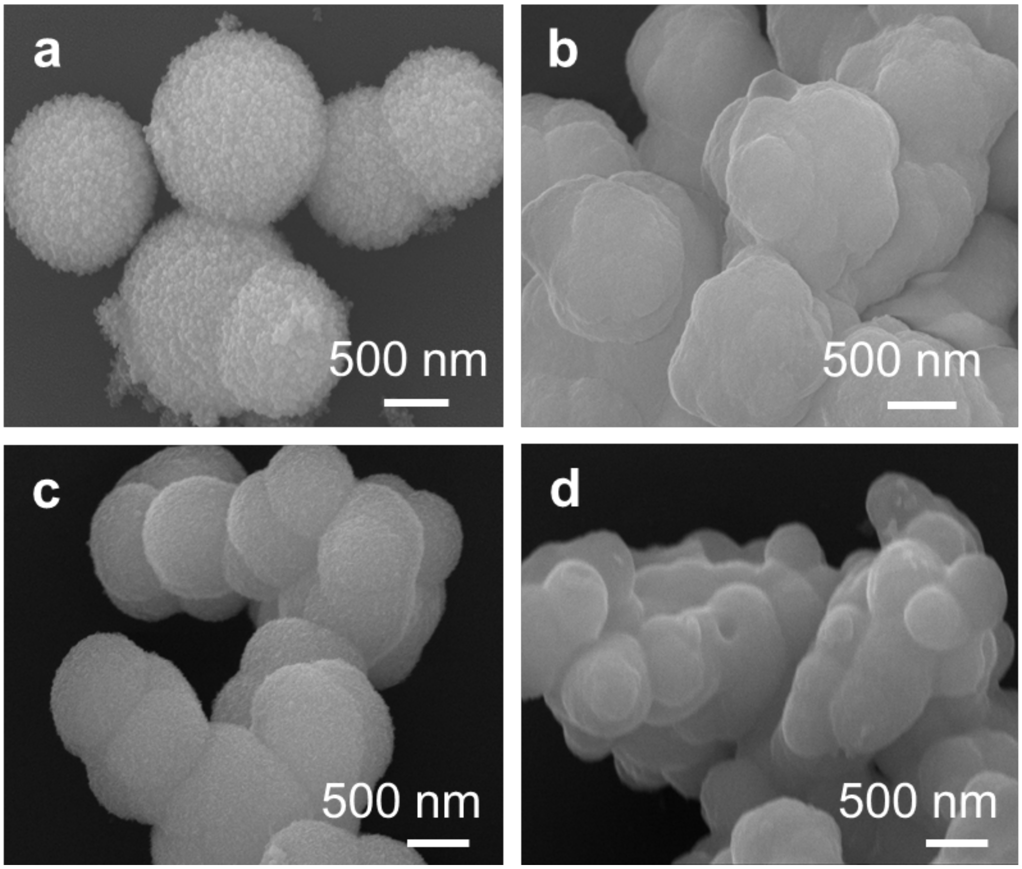

Supplementary Fig. 10 | SEM images of precatalysts for **(a)** CuP0, **(b)** CuP0.2, **(c)** CuP0.4, and **(d)** CuP0.6.

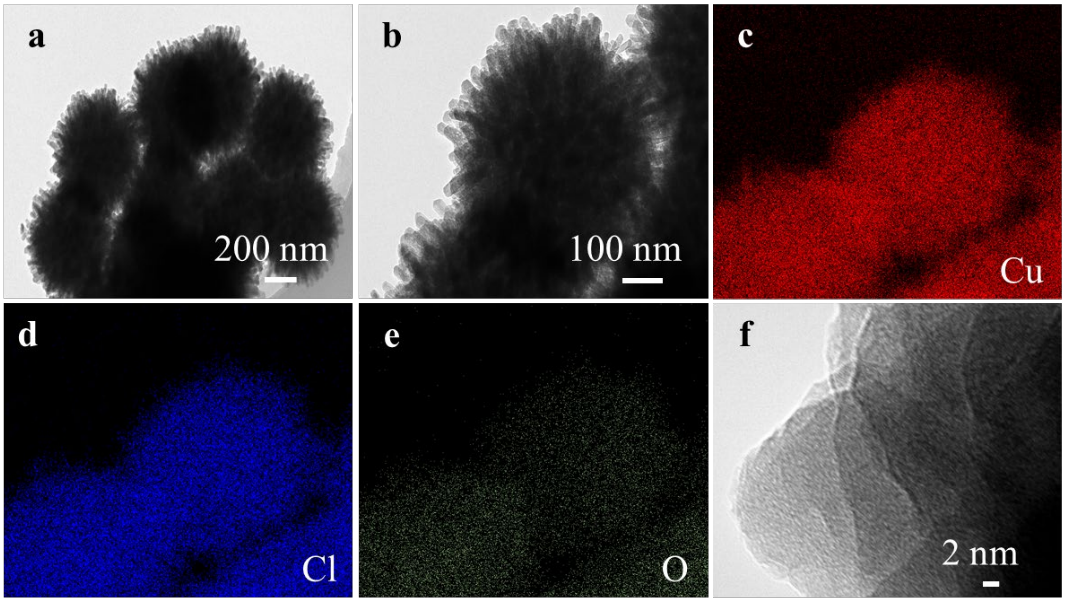

Supplementary Fig. 11 | Characterization of the CuP0 precatalyst without phosphate doping. a,b, TEM images with different magnifications. **c-e,** Corresponding element mappings of **(c)** Cu, **(d)** Cl, and **(e)** O. **f,** HRTEM image.

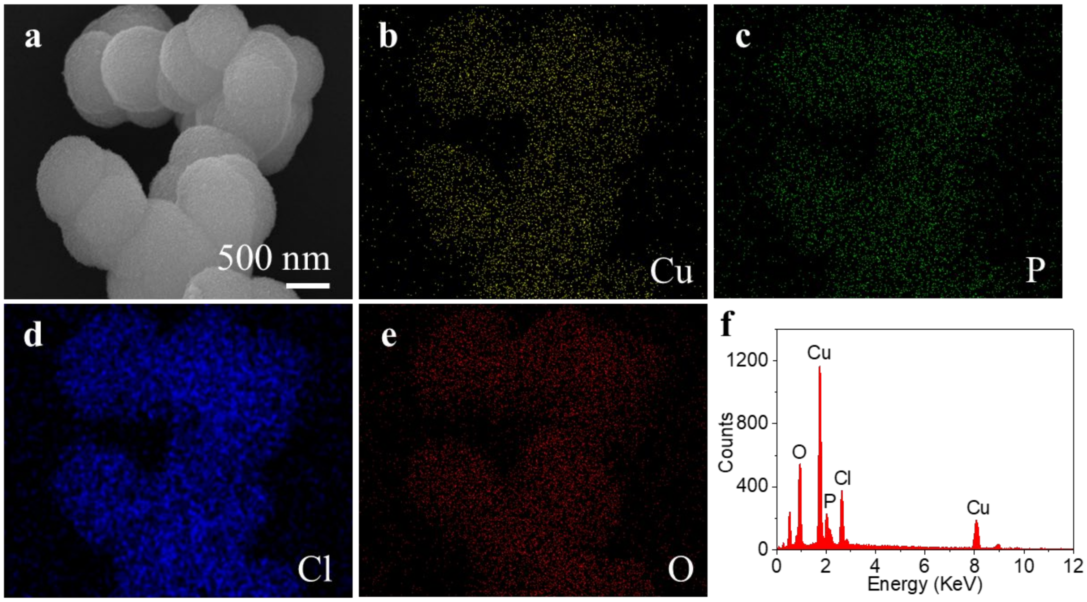

Supplementary Fig. 12 | Characterization of the CuP0.4 precatalyst. a, SEM image. **b-e,** Corresponding element mappings of **(b)** Cu, **(c)** P, **(d)** Cl, and **(e)** O. **f,** EDX spectrum.

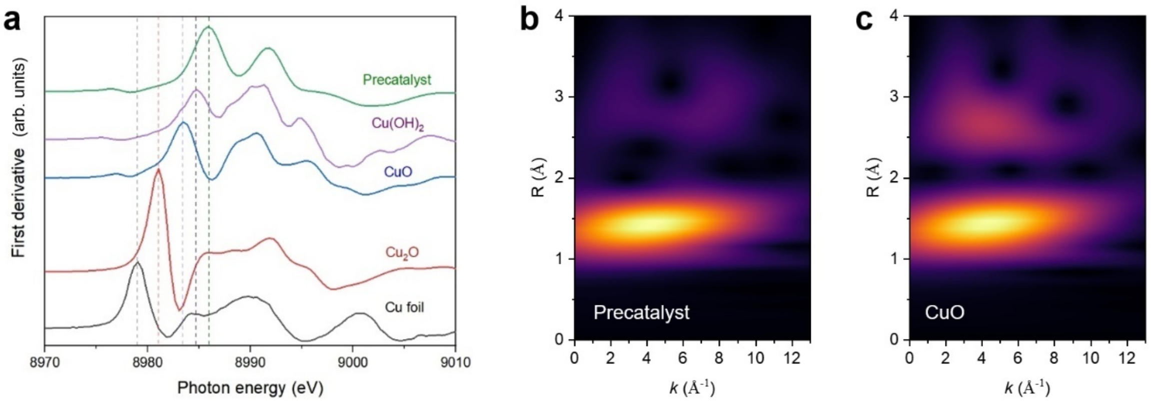

Supplementary Fig. 13 | a, The Cu K-edge XANES derivative spectra of Cu precatalyst and standards (Cu foil, Cu_2O, CuO, and $Cu(OH)_2$), in which the blueshift of XANES derivative maximum in the precatalyst is due to the ligand effect from phosphate doping. **b,c,** Wavelet transform of the Cu K-edge EXAFS of (**b**) precatalyst and (**c**) CuO standard.

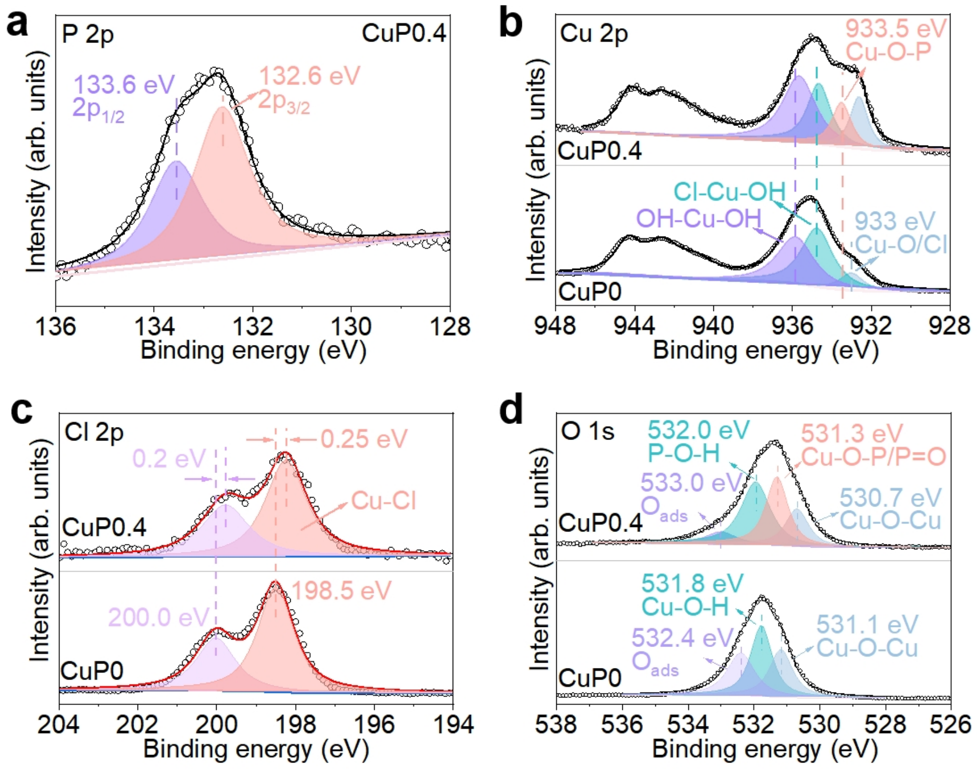

Supplementary Fig. 14 | High-resolution XPS spectra of **(a)** P 2p, **(b)** Cu 2p, **(c)** Cl 2p, and **(d)** O 1s for CuP0.4 and CuP0 precatalysts.

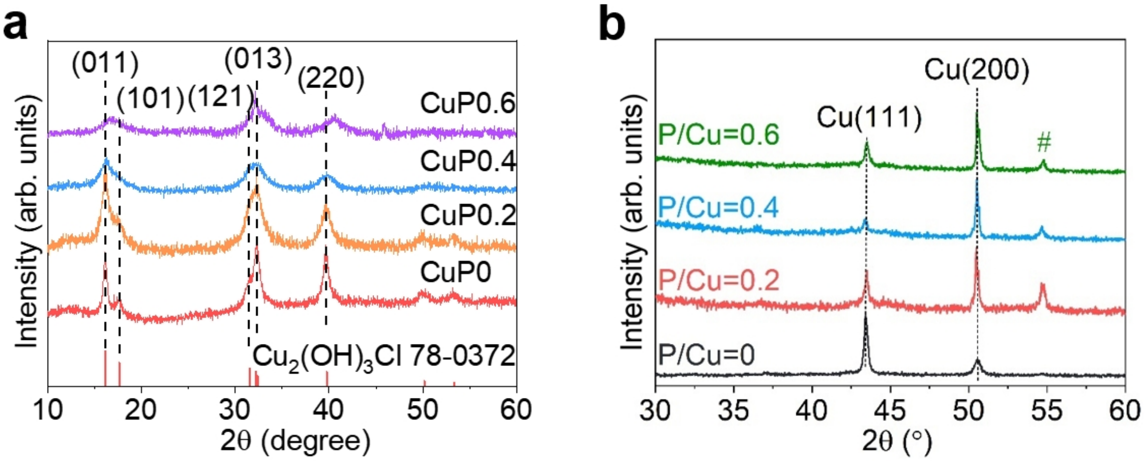

Supplementary Fig. 15 | a, XRD patterns for CuP0, CuP0.2, CuP0.4, and CuP0.6 precatalysts. **b,** XRD patterns of different Cu catalysts derived from various phosphate-loaded precatalysts. # represents the diffraction peak of carbon paper substrate.

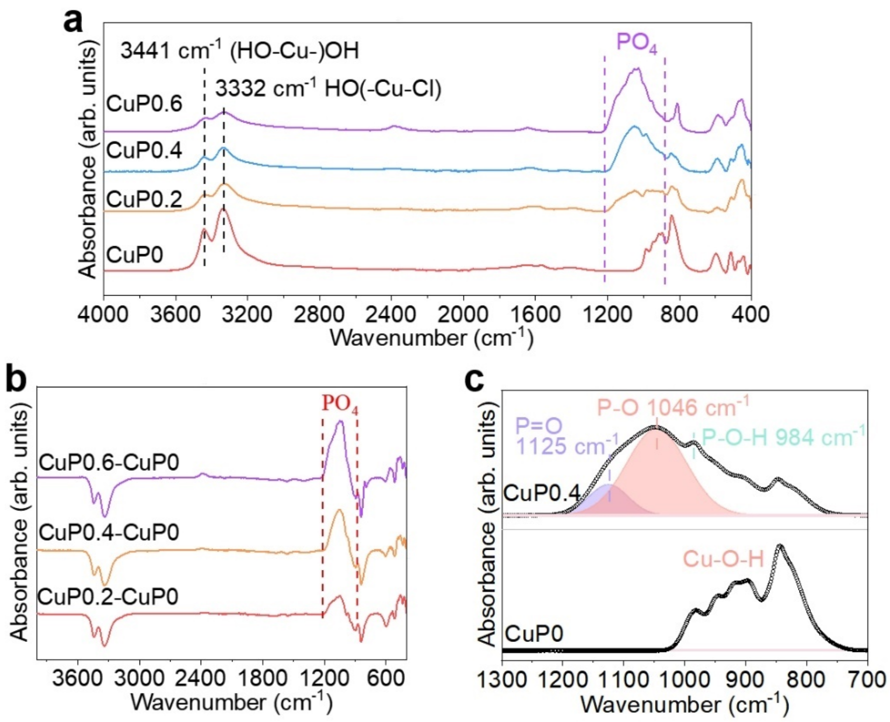

Supplementary Fig. 16 | FTIR spectra for precatalysts. a, FTIR spectra for CuP0, CuP0.2, CuP0.4, and CuP0.6 precatalysts. **b,** The difference spectra between the different P-doping concentration precatalysts with CuP0 precatalyst. **c,** FTIR spectra for CuP0.4 and CuP0 precatalysts in the range of 1300~700 cm^{-1} and the corresponding fitting spectra of CuP0.4 precatalyst.

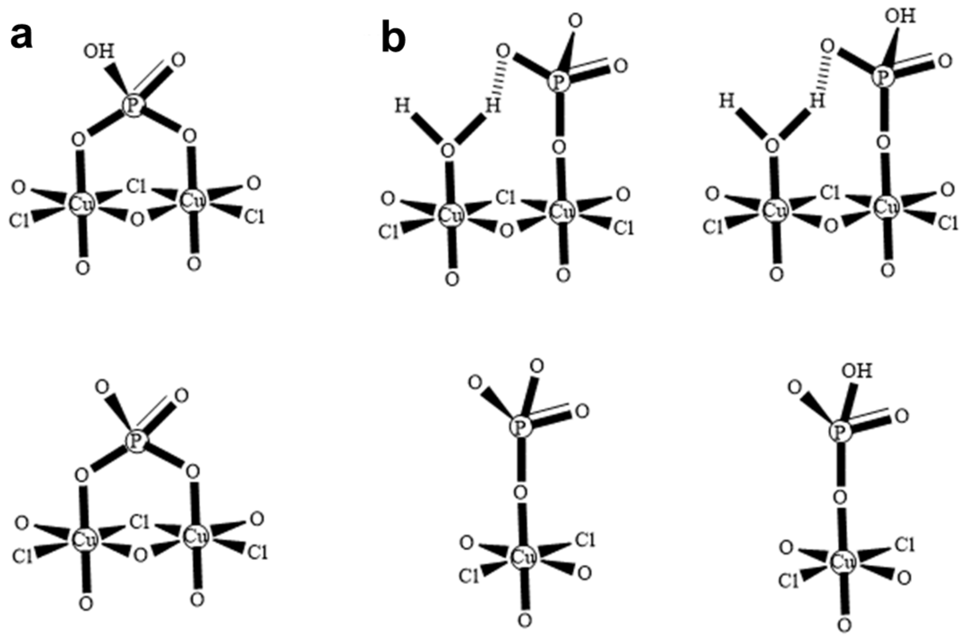

Supplementary Fig. 17 | Possible geometry structures of **(a)** bidentate and **(b)** monodentate ligands in various phosphate-copper complexes.

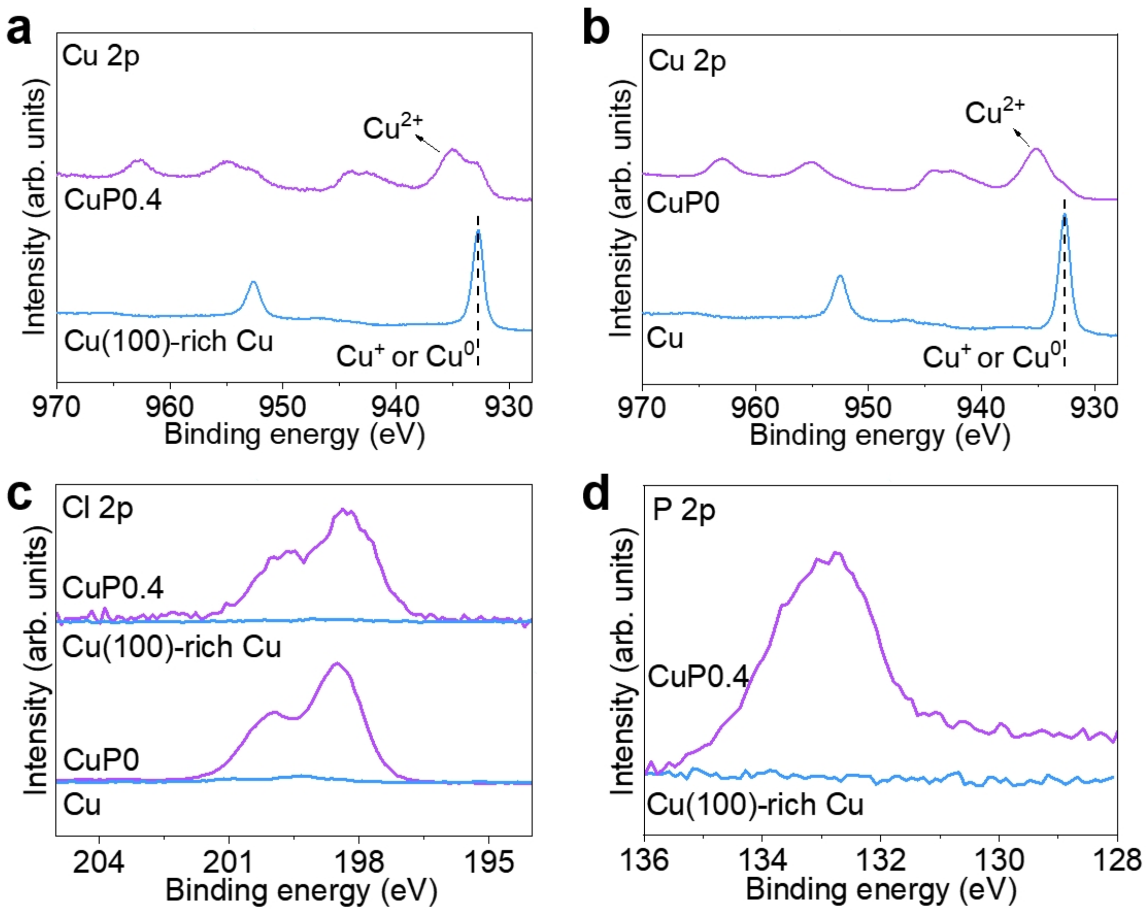

Supplementary Fig. 18 | High-resolution XPS spectra. a, Cu 2p for CuP0.4 and Cu(100)-rich catalyst. **b,** Cu 2p for CuP0 and Cu. **c,** Cl 2p for CuP0.4, Cu(100)-rich catalyst, CuP0, and Cu. **d,** P 2p for CuP0.4 and Cu(100)-rich catalyst. The results show the removal of nonmetallic elements and the reduction of Cu ions during the CO_2R process to form the active Cu sites.

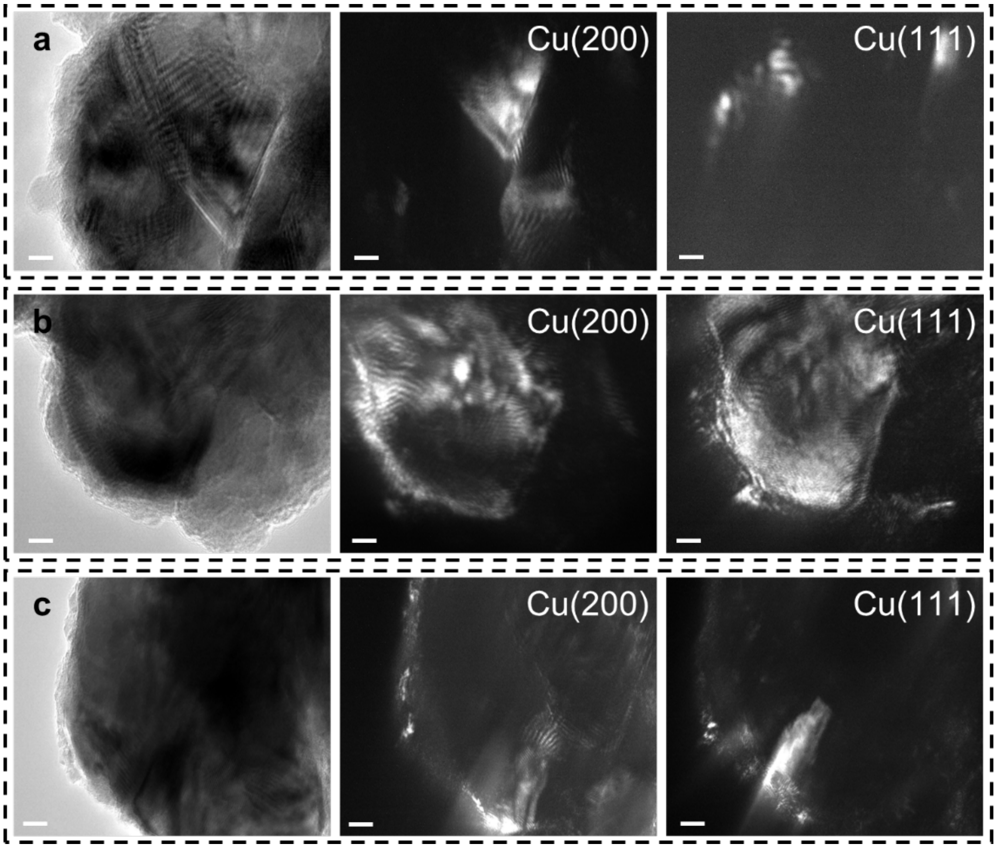

Supplementary Fig. 19 | High-resolution and dark-field TEM images at three different random sites on the Cu(100)-rich catalyst. The scale bars are 5 nm for **(a)** and **(b)**, and 10 nm for **(c)**.

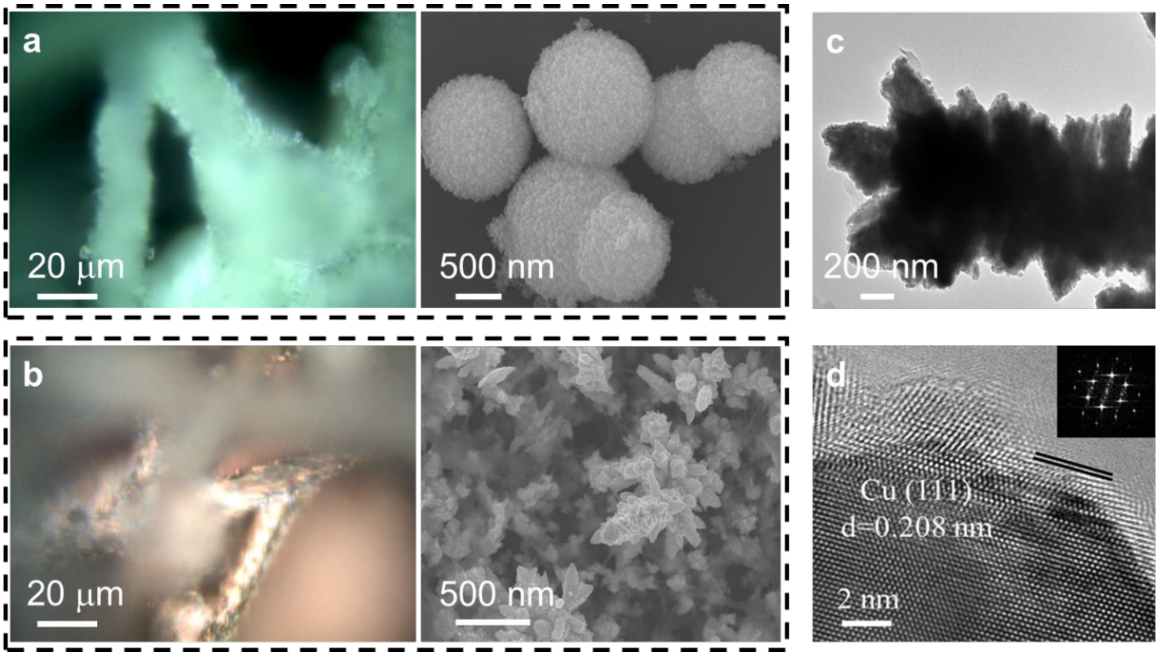

Supplementary Fig. 20 | a,b, Dark-field microscope images (left) and related SEM images (right) of CuP0 precatalyst after electrolysis at different reduction times, i.e. **(a)** 0 min and **(b)** 30 min. **c,d,** TEM images for Cu catalyst, the inset in **(d)** is the corresponding FFT pattern.

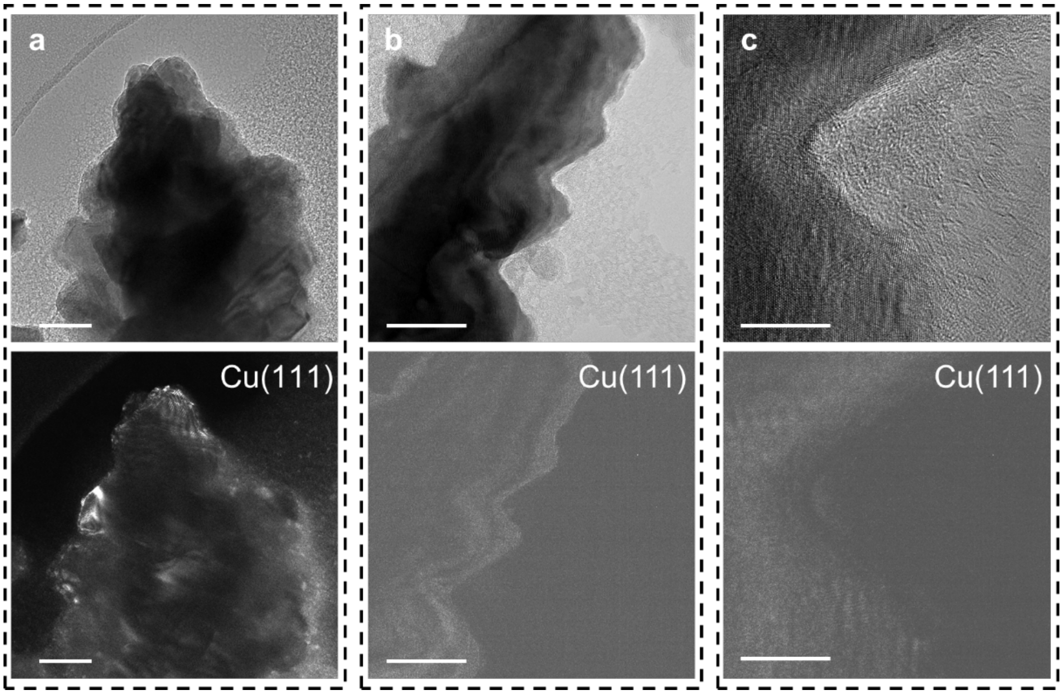

Supplementary Fig. 21 | High-resolution and dark-field TEM images for the Cu catalyst at three different random sites. The scale bars are 50 nm for **(a)** and **(b)**, and 10 nm for **(c)**.

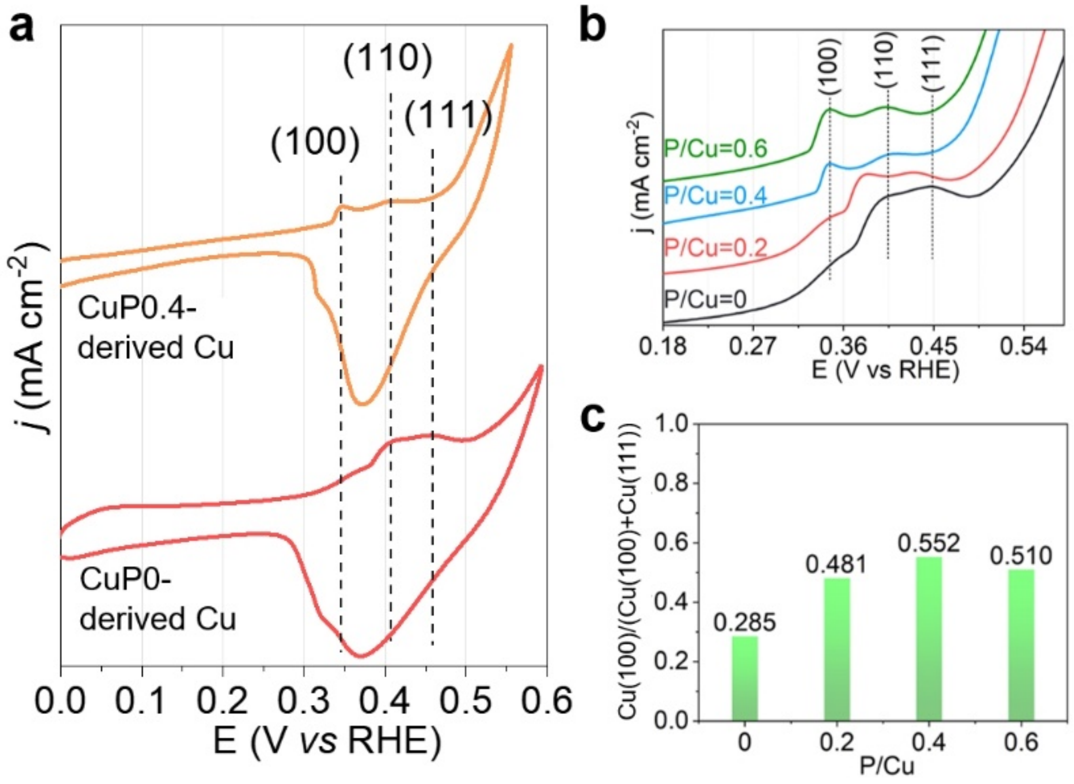

Supplementary Fig. 22 | **a,** CV curves collected in N_2-purged 1.0 M KOH for Cu(100)-rich and Cu(111)-rich catalysts. **b,** CV curves collected in N_2-purged 1.0 M KOH for different Cu catalysts derived from various phosphate-loaded precatalysts. **c,** The corresponding Cu(100)/(Cu(100)+Cu(111)) ratio obtained from the CV curves.

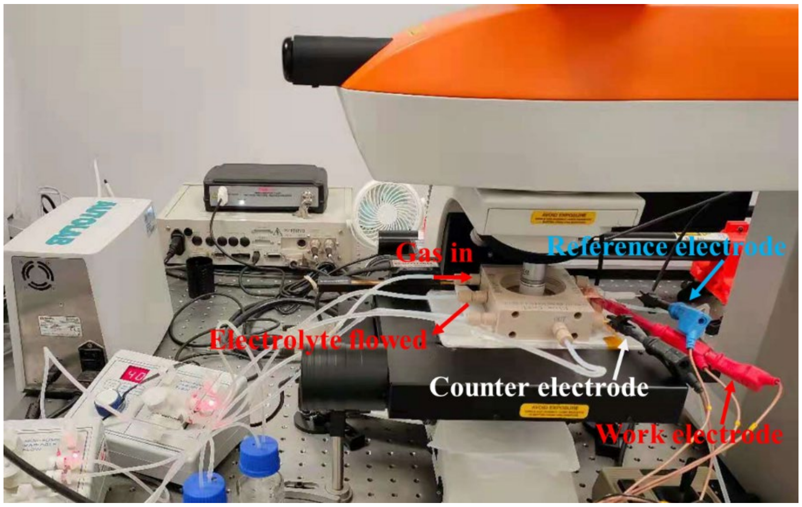

Supplementary Fig. 23 | A photograph of the *in-situ* Raman setup.

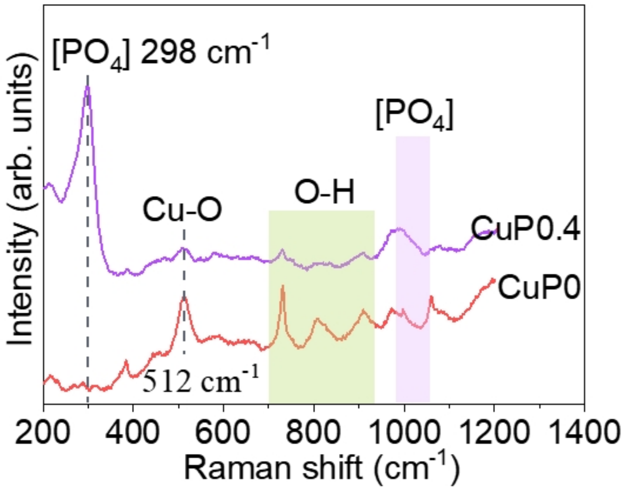

Supplementary Fig. 24 | Raman spectra of CuP0 and CuP0.4 precatalysts in 0.1 M $KHCO_3$ electrolyte at open circuit potential. For the CuP0 precatalyst, the lower frequency peak at 512 cm^{-1} was assigned to the Cu-O bond, which became widened after P-doping, likely due to the symmetric bending mode of PO_4 (ν_2) at 472 cm^{-1}. For the CuP0.4 precatalyst, P-O vibrations in phosphate anions showed the antisymmetric stretching mode (ν_3) at 1019 cm^{-1}, the symmetric stretching mode (ν_1) at 998 cm^{-1}, and the peak at 298 cm^{-1} attributable to the [PO_4] species in reichenbachite and libethenite[1].

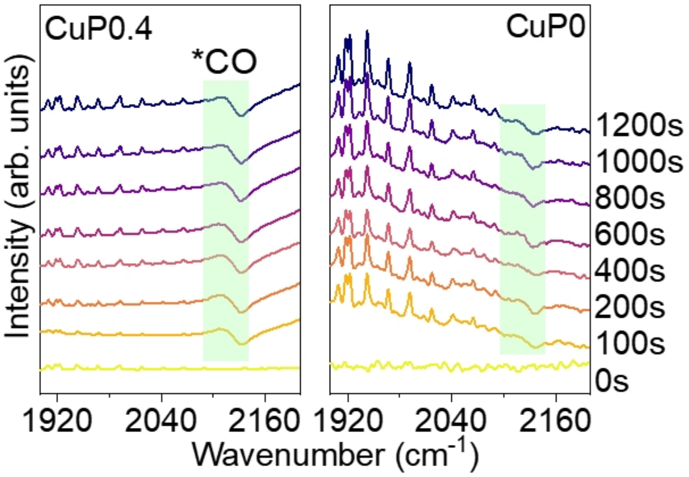

Supplementary Fig. 25 | Time-dependent *in-situ* ATR-SEIRAS spectra for CuP0.4 (left) and CuP0 (right) precatalysts.

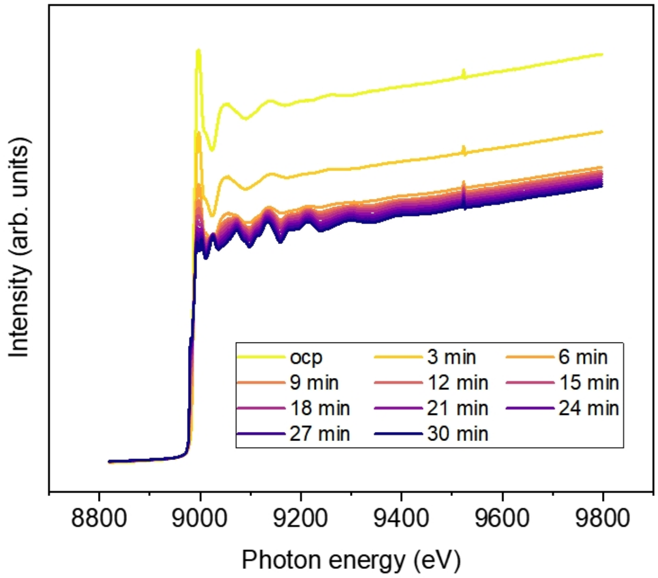

Supplementary Fig. 26 | Time-dependent Cu K-edge XAFS spectra (before normalization) of Cu precatalyst with phosphate addition, in which the edge jump of XAFS spectrum drastically decreases at the first 15 mins and then remains steady at the later stage, indicating that the catalyst is dissolved and re-deposited. The test was performed in CO_2-flowed 0.1 M $KHCO_3$ electrolyte at -1.1 V *vs* RHE over the course of 30-min reduction time; ocp stands for open-circuit potential.

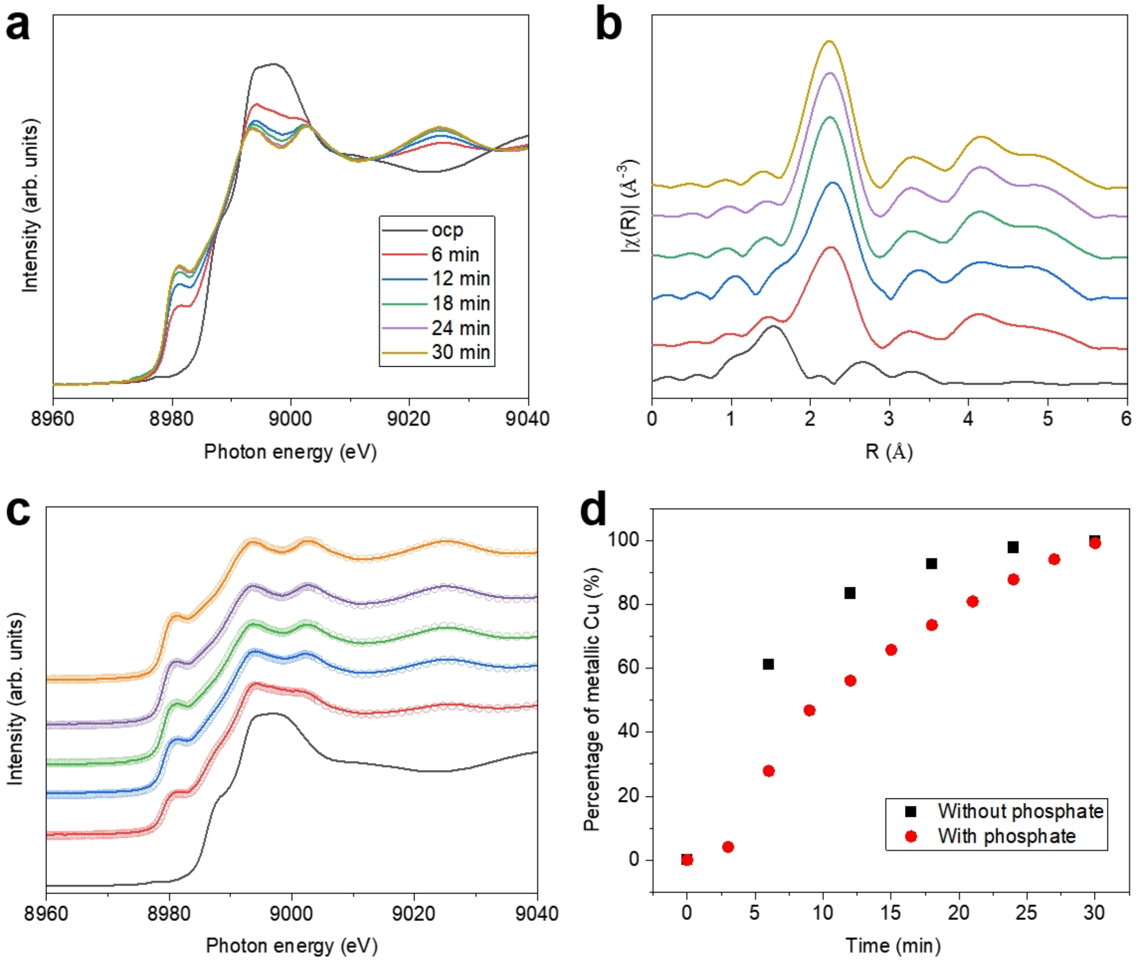

Supplementary Fig. 27 | a, b, Time-dependent Cu K-edge XANES (**a**) and Fourier-transformed EXAFS (**b**) of control Cu precatalyst without phosphate, the test was performed in CO_2-flowed 0.1 M $KHCO_3$ electrolyte at -1.1 V *vs* RHE over the course of 30-min reduction time; ocp stands for open-circuit potential. **c,** The corresponding XANES fitting spectra in (**a**), solid lines represent the experiment data and circles represent the linear combination fit spectra. **d,** The percentage of metallic Cu at different reduction times for Cu precatalyst with phosphate and control Cu precatalyst without phosphate. Values are extracted from linear combination fit using the XANES spectra of Cu precatalyst at ocp and metallic Cu with the weighting factors as fit parameters.

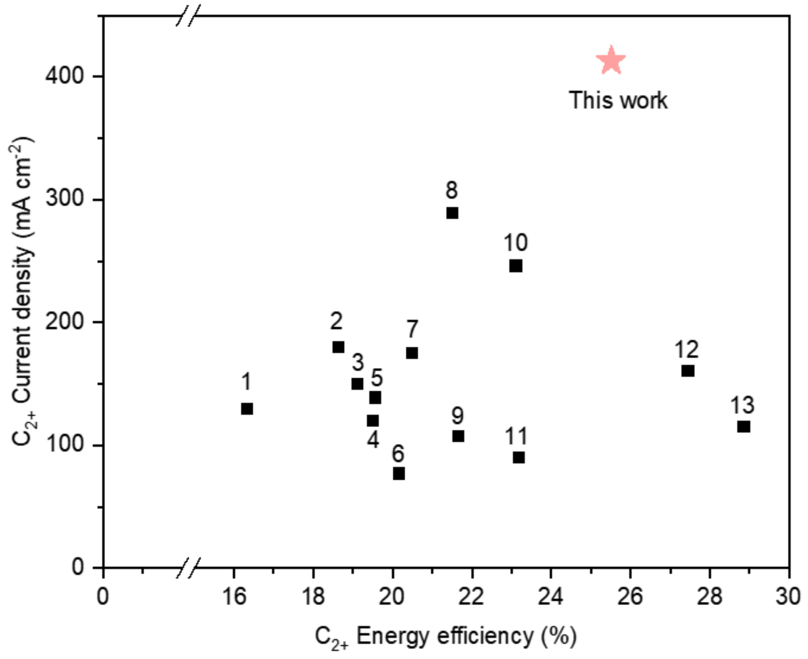

Supplementary Fig. 28 | Performance comparison for the Cu-based catalysts tested in similar MEA-CO_2R systems using bicarbonate electrolytes. Details are included in Supplementary Table 11. References: 1 for [2], 2 for [3], 3 for [4], 4 for [5], 5 for [6], 6 for [7], 7 for [8], 8 for [9], 9 for [10], 10 for [11], 11 for [12], 12 for [13], and 13 for [14].

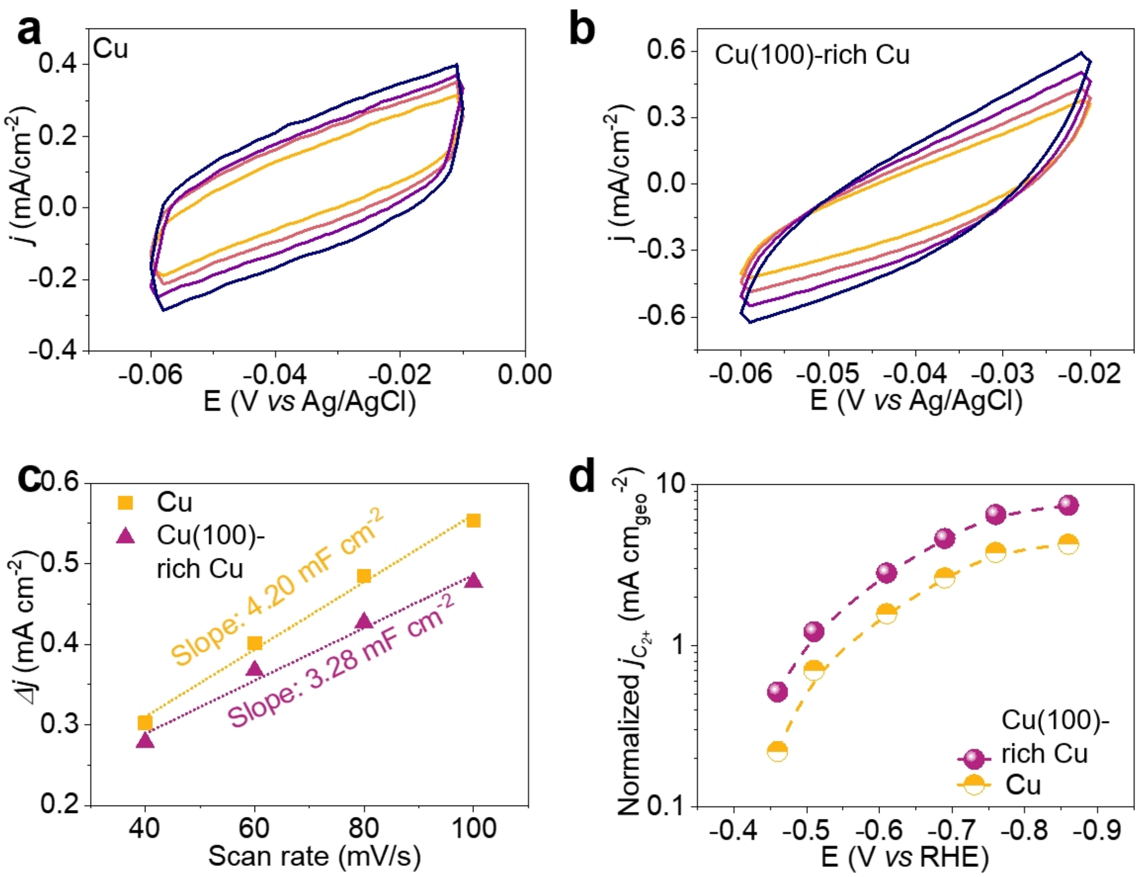

Supplementary Fig. 29 | a,b, CV curves collected in N_2-saturated 1.0 M KOH with scan rates from 40 mV/s to 100 mV/s for **(a)** Cu catalyst and **(b)** Cu(100)-rich catalyst. **c,** Correlation between the absolute current density difference and the scan rate, double-layer capacitance (C_{dl}) is equal to half of the linear slope. **d,** ECSA-normalized C_{2+} current densities for Cu(100)-rich catalyst and Cu catalysts.

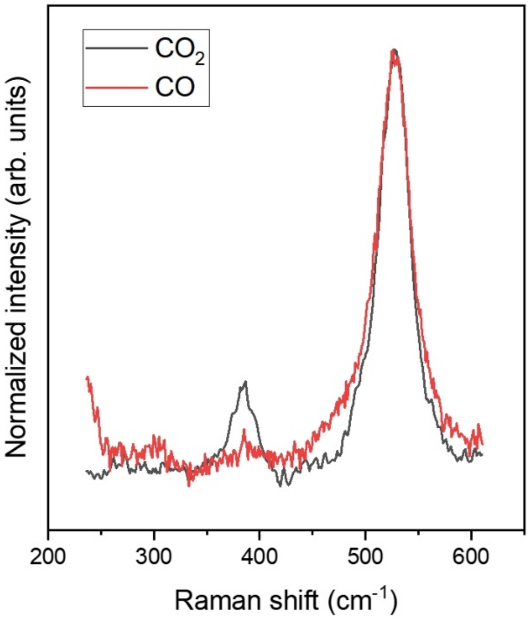

Supplementary Fig. 30 | Comparison of *in-situ* Raman spectra for Cu(100)-rich catalyst derived from CO_2 electrolysis in 0.1 M $KHCO_3$ and CO electrolysis in 1 M KOH. The CO_2-related spectrum has a higher *CO / OH^- ratio (correlated to the peak intensity ratio of ~380 cm^{-1} and ~520 cm^{-1}) than the CO-related spectrum.

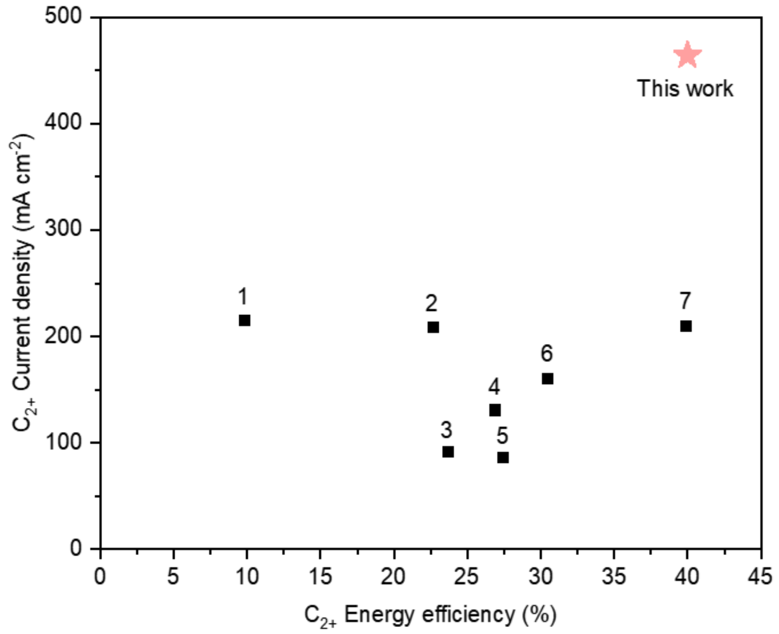

Supplementary Fig. 31 | Comparison of stable COR catalytic performance of various Cu-based catalysts in similar alkaline MEA systems at stabilities > 100 hours. Details are included in Supplementary Table 13. References: 1 for [15], 2 for [16], 3 for [17], 4 for [18], 5 for [19], 6 for [20], and 7 for [21].

Supplementary Table 1 | Calculated surface energies for Cu(100) and Cu(111) facets at different coverages of CO* and OH^-.

Facet	CO* coverage	Surface energy (J/m^2)	Facet	OH^- coverage	Surface energy (J/m^2)
Cu(111)	0	1.328	Cu(111)	0	1.328
Cu(111)	1/9 ML	1.268	Cu(111)	1/9 ML	1.110
Cu(111)	2/9 ML	1.216	Cu(111)	2/9 ML	1.023
Cu(111)	3/9 ML	1.180	Cu(111)	3/9 ML	1.024
Cu(111)	4/9 ML	1.140	Cu(111)	4/9 ML	0.897
Cu(100)	0	1.472	Cu(100)	0	1.472
Cu(100)	1/9 ML	1.411	Cu(100)	1/9 ML	1.195
Cu(100)	2/9 ML	1.352	Cu(100)	2/9 ML	1.031
Cu(100)	3/9 ML	1.295	Cu(100)	3/9 ML	0.945
Cu(100)	4/9 ML	1.245	Cu(100)	4/9 ML	0.634

Supplementary Table 2 | The dipole moment of Cu(100) and Cu(111) with different OH^- coverage.

Dipole moment (electrons × Angstrom)	1/9 ML	2/9 ML
Cu(100)	-0.491883	-0.491883

Supplementary Table 3 | Calculation of work function of Cu(100) with 1/9 ML and 2/9 ML of OH^- coverage.

OH^- coverage	1/9 ML	2/9 ML
E-fermi (eV)	1.8345	1.8345
Electrostatic potential energy (eV)	4.94017	4.94017
Work Function (eV)	3.10567	3.10567

Supplementary Table 4 | The energy of 1/9 ML of OH^{-} coverage on Cu(100) with/without spin polarization.

	System Energy (eV)
with spin polarization	-132.81613
without spin polarization	-132.81613

Supplementary Table 5 | Calculated area proportion of Cu(100) and Cu(111) facets at different coverages of CO* and OH^- from the Wulff construction analysis.

**Cu(111)**		**CO* coverage**				
		0	1/9 ML	2/9 ML	3/9 ML	4/9 ML
**OH^- coverage**	0	0.732	0.734	0.729	0.726	0.724
	1/9 ML	0.716	0.718	0.717	0.709	0.708
	2/9 ML	0.684	0.712	0.685	0.673	0.668
	3/9 ML	0.637	0.638	0.635	0.623	0.583
	4/9 ML	0.518	0.517	0.507	0.488	0.478
**Cu(100)**		**CO* coverage**				
		0	1/9 ML	2/9 ML	3/9 ML	4/9 ML
**OH^- coverage**	0	0.268	0.266	0.271	0.274	0.276
	1/9 ML	0.284	0.282	0.283	0.291	0.292
	2/9 ML	0.316	0.288	0.315	0.327	0.332
	3/9 ML	0.363	0.362	0.365	0.377	0.417
	4/9 ML	0.482	0.483	0.493	0.512	0.522

Supplementary Table 6 | Atomic percentages of different elements in precatalysts as measured by EDX and a comparison of the P/Cu atomic ratios extracted from EDX and ICP-OES.

**At%**	**Cu**	**O**	**Cl**	**P**	**P/Cu atomic ratio**	
					EDX	ICP-OES
Cu P0	36.17	47.59	16.24	0	0	0
CuP0.2	31.62	49.98	12.84	5.56	0.175	0.2
CuP0.4	27.96	52.90	8.13	11.01	0.394	0.4
CuP0.6	25.14	53.17	8.01	13.68	0.544	0.6

Supplementary Table 7 | Comparison of this work with a previous work[3] that reported the in situ growth of Cu(100) using *CO.

		This work	Ref.[3]
**Catalyst synthesis**		In situ reduction of phosphate-doped precatalyst	Electrodeposition of Cu from Cu(II) ditartrate
**Cu(100) promoter**		*CO&OH$^-$	*CO
**Cu(100)/(Cu(111)+Cu(100))**		0.684	0.287†
**MEA-CO_2R performance**	**FE_{C2+}**	83%	60%
	**j_{C2+}**	415 mA cm^{-2}	180 mA cm^{-2}
	**EE_{C2+}**	25.5%	18.6%
**MEA-COR performance**	**FE_{C2+}**	93%	not available
	**j_{C2+}**	465 mA cm^{-2}	not available
	**EE_{C2+}**	37%	not available
	**$SPCE_{C2+}$**	95%	not available

†This value is calculated based on the XRD pattern of Cu-CO_2 catalyst (63s) reported in Supplementary Fig. 18a of Ref. [3].

Supplementary Table 8 | The Cu(100)/(Cu(100)+Cu(111)) ratio of different Cu catalysts derived from various phosphate-loaded precatalysts calculated from the XRD patterns in Supplementary Fig. 15b.

P/Cu ratio in precatalyst	Area of Cu(111)	Area of Cu(100)	Cu(100)/(Cu(100)+Cu(111))
0.6	0.1447	0.1969	0.577
0.4	0.0916	0.1979	0.684
0.2	0.1999	0.2156	0.416
0	0.3221	0.1365	0.298

Supplementary Table 9 | Linear combination fit analysis to obtain the percentages of the CuP0.4 precatalyst and metallic Cu at different reduction times.

Time	CuP0.4 precatalyst (%)	Cu metal (%)
0 min	1	0
3 min	95.9	4.1
6 min	72.1	27.9
9 min	53.1	46.9
12 min	43.8	56.2
15 min	34.2	65.8
18 min	26.2	73.6
21 min	19.0	71.0
24 min	12.1	87.9
27 min	5.8	94.2
30 min	0.8	99.2

Supplementary Table 10 | EXAFS fitting results for CuP0.4 during CO_2R over the course of 30-min reduction time. Data range k = 3-11 $Å^{-1}$, amplitude reduction factor S_0^2 = 0.8. We referred to a previous work[22] for the fitting principle. Briefly, the coordination numbers (N) were fixed to the expected values listed in cif files of $Cu_2(OH)_3Cl$ and Cu foil, bond distances (R) and the Debye Waller factor (σ^2) for each cell were determined. Then the Debye Waller values were fixed to calculate N. Numbers marked with * are fixed according to the information in the cif file. Bolded and unbolded scatter paths are from $Cu_2(OH)_3Cl$ and metallic Cu, respectively.

**Time**	**Scatter path**	**CN**	***R* (Å)**	**σ^2 ($Å^2$)**	**R_f**
0 min (ocp)	**Cu-O**	1.54±0.63	1.96±0.03	0.02536*	0.94%
	**Cu-O**	2.09±0.17	2.00±0.05	0.00269*	
	**Cu-Cl**	1.48±0.76	2.71±0.20	0.03381*	
	**Cu-Cu**	2.15±0.34	3.03±0.10	0.00799*	
	**Cu-Cu**	2.26±0.65	3.21±0.21	0.00854*	
	**Cu-Cu**	2.13±0.49	3.42±0.06	0.00750*	
3 min	**Cu-O**	1.14±0.78	1.95±0.04	0.02536*	0.58%
	**Cu-O**	2.28±0.22	1.98±0.03	0.00269*	
	Cu-Cu	1.53±0.23	2.50±0.06	0.00953*	
	**Cu-Cu**	2.44±0.69	3.00±0.13	0.00799*	
	**Cu-Cu**	2.28±0.49	3.22±0.19	0.00854*	
	**Cu-Cu**	2.37±0.84	3.44±0.04	0.00750*	
6 min	**Cu-O**	1.00±0.36	1.88±0.07	0.02536*	2.77%
	**Cu-O**	2.06±0.82	1.96±0.03	0.00269*	
	Cu-Cu	3.02±0.26	2.55±0.01	0.00953*	
	**Cu-Cu**	3.47±0.36	3.06±0.08	0.00799*	
	**Cu-Cu**	3.64±0.80	3.28±0.13	0.00854*	
	**Cu-Cu**	1.93±0.98	3.49±0.01	0.00750*	
9 min	**Cu-O**	0.95±0.73	1.88±0.07	0.02536*	1.25%

	**Cu-O**	1.31±0.41	1.94±0.05	0.00269*	
	Cu-Cu	4.52±0.28	2.55±0.01	0.00953*	
12 min	**Cu-O**	0.85±0.23	1.84±0.11	0.02536*	1.24%
	**Cu-O**	1.19±0.43	1.93±0.06	0.00269*	
	Cu-Cu	5.52±0.31	2.55±0.01	0.00953*	
15 min	**Cu-O**	0.78±0.25	1.85±0.10	0.02536*	1.23%
	**Cu-O**	0.91±0.47	1.92±0.07	0.00269*	
	Cu-Cu	6.46±0.35	2.55±0.01	0.00953*	
18 min	**Cu-O**	0.62±0.07	1.86±0.09	0.02536*	1.01%
	**Cu-O**	0.65±0.46	1.92±0.07	0.00269*	
	Cu-Cu	7.17±0.34	2.54±0.01	0.00953*	
21 min	**Cu-O**	0.45±0.19	1.89±0.10	0.02536*	0.81%
	**Cu-O**	0.45±0.39	1.92±0.03	0.00269*	
	Cu-Cu	7.95±0.33	2.54±0.01	0.00953*	
24 min	**Cu-O**	0.07±0.01	1.81±0.14	0.02536*	1.99%
	**Cu-O**	0.38±0.17	1.90±0.10	0.00269*	
	Cu-Cu	8.62±0.51	2.54±0.01	0.00953*	
27 min	**Cu-O**	0.20±0.04	1.88±0.10	0.00269*	1.80%
	Cu-Cu	9.33±0.45	2.54±0.01	0.00953*	
30 min	Cu-Cu	9.85±0.41	2.54±0.01	0.00953*	1.75%

Supplementary Table 11 | Performance comparison for the Cu-based catalysts tested in similar neutral MEA-CO_2R systems using bicarbonate electrolytes.

Catalyst	Electrolyte	E (V)	FE_{C2+} (%)	j_{C2+} ($mA\ cm^{-2}$)	EE_{C2+} (%)	Ref.
**Cu(100)-rich Cu**	**0.1 M $KHCO_3$**	**-3.79**	**83**	**413**	**25.5**	**This work**
Molecule/Cu	0.1 M $KHCO_3$	-3.65	64	77	20.2	[7]
N-doped C/Cu	0.2 M $KHCO_3$	-3.67	92	115	28.8	[14]
FeTTP[Cl]/Cu	0.1 M $KHCO_3$	-3.7	75	90	23.2	[12]
Electrodeposited Cu	0.15 M $KHCO_3$	-3.7	60	180	18.6	[3]
Sputtered Cu	0.1 M $KHCO_3$	-3.9	65	150	19.1	[4]
Dense vertical lamellate Cu	0.5 M $KHCO_3$	-3.35	80	160	27.6	[13]
CuSi	0.1 M $KHCO_3$	-4.1	82	246	23	[11]
Defect-site-rich Cu	0.1 M $KHCO_3$	-3.5	60	120	19.5	[5]
Catalyst/tetrahydro-phenantrolinium/ionomer Cu	0.1 M $KHCO_3$	-3.7	63	139	19.6	[6]
Core-shell Cu-C	0.1 M $KHCO_3$	-3.8	71	107	21.6	[10]
CeO_2/Cu	0.1 M $KHCO_3$	-3.7	68	289	21.4	[9]
MOF-augmented Cu	0.1 M $KHCO_3$	-4.0	70	175	20.5	[8]
Cu(OH)BTA	0.1 M $KHCO_3$	-3.8	54	130	16.3	[2]

Supplementary Table 12 | Double-layer capacitance and corresponding ECSA for Cu and Cu(100)-rich catalysts. The double-layer capacitance of electropolished Cu foil was obtained from the previous report[23].

Catalyst	Capacitance (mF cm^{-2})	ECSA (cm^2)
Cu	2.10	72
Cu(100)-rich Cu	1.64	56
Electropolished Cu foil	0.029	1

Supplementary Table 13 | Comparison of stable COR catalytic performance of various Cu-based catalysts in similar alkaline MEA systems at stabilities > 100 hours.

Catalyst	System	E (V)	FE_{C2+} (%)	j_{C2+} (mA cm^{-2})	EE_{C2+} (%)	$SPCE_{C2+}$ (%)	Stability (h)	Ref.
**Cu(100)-rich Cu**	**1 bar CO and 1 M KOH**	**-2.3**	**93**	**465**	**41**	**95**	**150**	**This work**
COF/Cu	1 bar CO and 1 M KOH	-2.23	87	210	40	95	200	[21]
Cu-Pd	1 bar CO and 1 M KOH	-3.4	43	215	10	27	500	[15]
Cu-in-Ag	10 bar CO and 2.5 M KOH	-2.5	86	86	27	9	820	[19]
CP-Cu	0.25 bar CO and 3.5 M KOH	-2.75	70	208	23	61	250	[16]
Cu	1 bar CO and 3 M KOH	-2.32	80	160	30	40	120	[20]
Cu-Ag	1 bar CO and 1 M KOH	-3.0	87	131	27	76	103	[18]
Cu:Py:SSC	1 bar CO and 3 M KOH	-2.73	61	91	24	43	110	[17]

References

1 Frost, R. L., Williams, P. A., Martens, W., Kloprogge, J. T. & Leverett, P. Raman spectroscopy of the basic copper phosphate minerals cornetite, libethenite, pseudomalachite, reichenbachite and ludjibaite. *J. Raman Spectrosc.* **33**, 260-263 (2002).

2 Liang, Y. *et al.* Stabilizing copper sites in coordination polymers toward efficient electrochemical CC coupling. *Nat. Commun.* **14**, 474 (2023).

3 Wang, Y. *et al.* Catalyst synthesis under CO_2 electroreduction favours faceting and promotes renewable fuels electrosynthesis. *Nat. Catal.* **3**, 98-106 (2020).

4 Gabardo, C. M. *et al.* Continuous carbon dioxide electroreduction to concentrated multi-carbon products using a membrane electrode assembly. *Joule* **3**, 2777-2791 (2019).

5 Gu, Z. *et al.* Efficient electrocatalytic CO_2 reduction to C_{2+} alcohols at defect-site-rich Cu surface. *Joule* **5**, 429-440 (2021).

6 Ozden, A. *et al.* High-rate and efficient ethylene electrosynthesis using a catalyst/promoter/transport Layer. *ACS Energy Lett.* **5**, 2811-2818 (2020).

7 Li, F. *et al.* Molecular tuning of CO_2-to-ethylene conversion. *Nature* **577**, 509-513 (2020).

8 Nam, D. H. *et al.* High-rate and selective CO_2 electrolysis to ethylene via metal–organic-framework-augmented CO_2 availability. *Adv. Mater.* **34**, 2207088 (2022).

9 Wang, H. *et al.* Strain in copper/ceria heterostructure promotes electrosynthesis of multicarbon products. *ACS Nano* **17**, 346-354 (2023).

10 Kim, J.-Y. *et al.* Quasi-graphitic carbon shell-induced Cu confinement promotes electrocatalytic CO_2 reduction toward C_{2+} products. *Nat. Commun.* **12**, 3765 (2021).

11 Li, J. *et al.* Silica-copper catalyst interfaces enable carbon-carbon coupling towards ethylene electrosynthesis. *Nat. Commun.* **12**, 2808 (2021).

12 Li, F. *et al.* Cooperative CO_2-to-ethanol conversion via enriched intermediates at molecule–metal catalyst interfaces. *Nat. Catal.* **3**, 75-82 (2019).

13 Liu, W. *et al.* Electrochemical CO_2 reduction to ethylene by ultrathin CuO nanoplate arrays. *Nat. Commun.* **13**, 1877 (2022).

14 Wang, X. *et al.* Efficient electrically powered CO_2-to-ethanol via suppression of deoxygenation. *Nat. Energy* **5**, 478-486 (2020).

15 Ji, Y. *et al.* Selective CO-to-acetate electroreduction via intermediate adsorption tuning on ordered Cu–Pd sites. *Nat. Catal.* **5**, 251-258 (2022).

16 Luo, M. *et al.* Coordination polymer electrocatalysts enable efficient CO-to-acetate conversion. *Adv. Mater.*, 2209567 (2023).

17 Ozden, A. *et al.* Cascade CO_2 electroreduction enables efficient carbonate-free production of ethylene. *Joule* **5**, 706-719, (2021).

18 Li, J. *et al.* Weak CO binding sites induced by Cu–Ag interfaces promote CO electroreduction to multi-carbon liquid products. *Nat. Commun.* **14**, 698 (2023).

19 Jin, J. *et al.* Constrained C_2 adsorbate orientation enables CO-to-acetate electroreduction. *Nature* **617**, 724-729 (2023).
20 Overa, S. *et al.* Enhancing acetate selectivity by coupling anodic oxidation to carbon monoxide electroreduction. *Nat. Catal.* **5**, 738-745 (2022).
21 Ozden, A. *et al.* Energy- and carbon-efficient CO_2/CO electrolysis to multicarbon products via asymmetric ion migration–adsorption. *Nat. Energy* **8**, 179-190 (2023).
22 Wang, X. *et al.* Morphology and mechanism of highly selective Cu(II) oxide nanosheet catalysts for carbon dioxide electroreduction. *Nat. Commun.* **12**, 794 (2021).
23 Li, C. W., Ciston, J. & Kanan, M. W. Electroreduction of carbon monoxide to liquid fuel on oxide-derived nanocrystalline copper. *Nature* **508**, 504-507 (2014).
